# Roles of NAD^+^ and Its Metabolites Regulated Calcium Channels in Cancer

**DOI:** 10.3390/molecules25204826

**Published:** 2020-10-20

**Authors:** Peilin Yu, Xiaobo Cai, Yan Liang, Mingxiang Wang, Wei Yang

**Affiliations:** 1 Department of Toxicology, and Department of Medical Oncology of Second Affiliated Hospital, Zhejiang University School of Medicine, Hangzhou 310058, Zhejiang, China; yupeilin@zju.edu.cn (P.Y.); liangy14@lzu.edu.cn (Y.L.); 2 Department of Biophysics, and Department of Neurosurgery of the First Affiliated Hospital, Zhejiang University School of Medicine, Hangzhou 310058, Zhejiang, China; caixb@zju.edu.cn; 3 BrioPryme Biologics, Inc., Hangzhou 310058, Zhejiang, China; mingxiang@briopryme.com

**Keywords:** NAD^+^, metabolites, calcium channels, cancer

## Abstract

Nicotinamide adenine dinucleotide (NAD^+^) is an essential cofactor for redox enzymes, but also moonlights as a regulator for ion channels, the same as its metabolites. Ca^2+^ homeostasis is dysregulated in cancer cells and affects processes such as tumorigenesis, angiogenesis, autophagy, progression, and metastasis. Herein, we summarize the regulation of the most common calcium channels (TRPM2, TPCs, RyRs, and TRPML1) by NAD^+^ and its metabolites, with a particular focus on their roles in cancers. Although the mechanisms of NAD^+^ metabolites in these pathological processes are yet to be clearly elucidated, these ion channels are emerging as potential candidates of alternative targets for anticancer therapy.

## 1. Introduction

**Figure 1:**
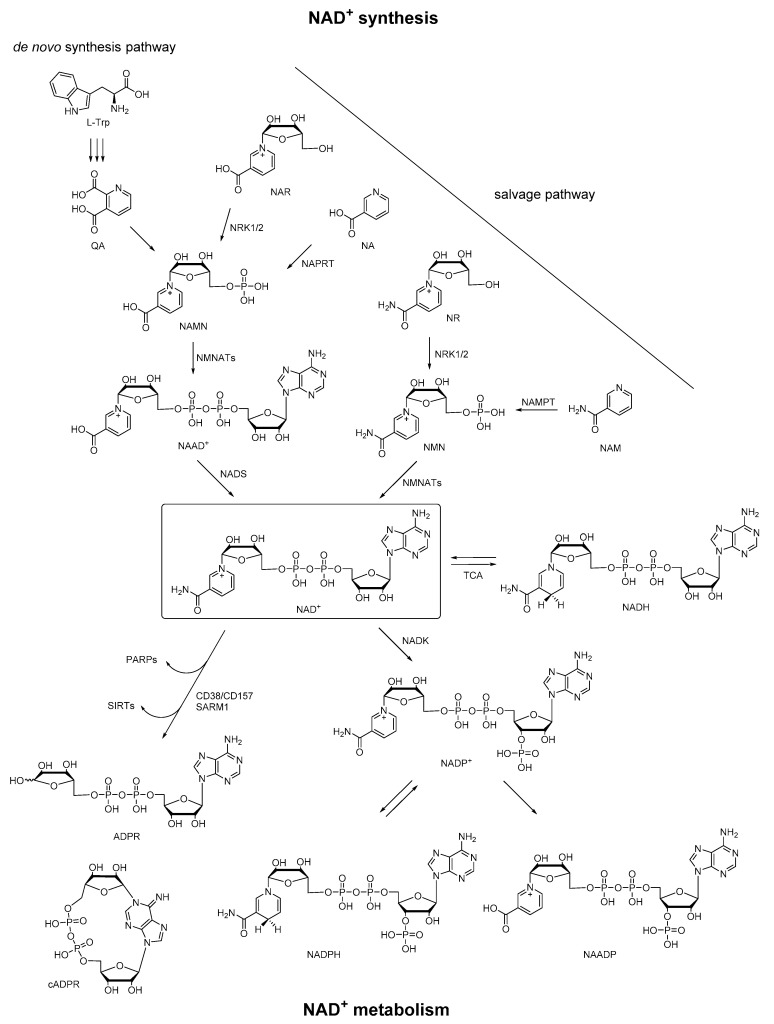
Pathways of NAD^+^ synthesis and metabolism.

Nicotinamide adenine dinucleotide (NAD^+^) is an essential biomolecule involved in many critical processes, especially in energy metabolism and electron transfer. Most organisms synthesize NAD^+^ via two major pathways: (1) synthesis from tryptophan (Trp) (the de novo pathway); (2) synthesis from various extracellular and intracellular precursors including nicotinic acid (NA), NA riboside (NAR), nicotinamide (NAM), and NAM riboside (NR) (the salvage pathway) [1]. In particular, in the de novo pathway, synthesis of NAD^+^ begins from the conversion of dietary precursor Trp to quinolinic acid (QA), which is converted to nicotinamide adenine mononucleotide (NAMN). NAMN is also synthesized in the salvage pathway via NAR by nicotinamide riboside kinases 1 and 2 (NRK1/2) or indirectly via NA by nicotinate phosphoribosyltransferase (NAPRT). NAMN is adenylated to nicotinic acid adenine dinucleotide (NAAD^+^) via nicotinamide mononucleotide adenyltransferases 1–3 (NMNATs) before NAD^+^ synthesis via NAD synthetase (NADS). Moreover, NAD^+^ can also be generated from nicotinamide mononucleotide (NMN) via NMNATs, and NMN is synthesized in a salvage pathway via NR by NRK1/2 or NAM by nicotinamide phosphoribosyltransferase (NAMPT). In the metabolome of NAD^+^, it is reduced by tricarboxylic acid (TCA) cycle enzymes to NADH, and can be regenerated from NADH. Besides, NAD^+^ can be converted to NADP^+^ by NAD^+^ kinase (NADK). The corresponding phosphorylated redox pair NADP/NADPH is a crucial TCA cycle intermediate that provides reducing equivalents for endogenous antioxidant defense systems to maintain redox homeostasis. Then NAD^+^ can be consumed by poly (ADP-ribose) polymerases (PARPs) and sirtuins (SIRTs), or be degraded by membrane-bound glycohydrolases CD38/CD157 or sterile α and TIR motif-containing 1 (SARM1) to cyclic ADP-ribose (cADPR) and ADPR [2,3,4,5,6]. In this enzymatic conversion of NAD^+^ to cADPR, the nicotinamide group of NAD^+^ is cleaved, and the N1 of the adenine is linked back to the terminal ribose, forming a head to tail circled molecule, which had been documented as a natural metabolite in a wide range of endogenous systems. On the other hand, NADP^+^ can also be converted to nicotinic acid adenine dinucleotide phosphate (NAADP) under the functions of the same set of enzymes, in which the structural change is the conversion of the amide group of the nicotinamide in NADP^+^ to a carboxyl group. Taken together, these pyridine nucleotides (NAAD^+^, NAD^+^, NADH, NADP^+^, NADPH, cADPR, ADPR, and NAADP) derived from NAD^+^ constitute a regulatory system of energy metabolism (Figure 1), which plays an important role in a variety of physiological and pathological processes [7].

As for energy metabolism, all living organisms need to trap or liberate energy or to synthesize essential cell constituents and metabolites. These processes are usually realized by oxidation–reduction reactions, which are accomplished by specialized biomolecules that package and shuttle energy between different processes. As we know, ATP is the universal energy currency of cells. However, to generate ATP, energy must be extracted from nutrients by a series of coupled catabolic reactions. This process requires specialized electron carriers that can deliver energy to the mitochondrial electron-transport chain. NAD^+^ and its metabolites would be one of the most important systems that accomplish the shuttling of electrons between different reactions, by which the redox homeostasis and bioenergetics of organisms are maintained [7]. As the soluble electron carriers, NAD(H) is recognized by enzymes that catalyze catabolic reactions of glycolysis and by components of the electron transport chain, and NADP(H) is recognized by enzymes that are involved primarily in anabolic (reductive) reactions, such as lipid or cholesterol synthesis or fatty acid chain elongation [8]. Generally, a high NAD^+^:NADH ratio is maintained to readily accept electrons generated by catabolic reactions, whereas the low NADP^+^:NADPH ratio reflects a state of readiness to donate electrons to biosynthetic reactions or antioxidant defense [9]. As a result, the availability and the redox state of NAD^+^ and its metabolites regulate the activity of the processes involved in the intermediary metabolism, biosynthesis, and antioxidant defense.

Cancer is a multistep progression, and its critical hallmark is the reprogramming of the energy metabolism, mainly reflected in the altered mitochondrial bioenergetic and biosynthetic state of the cancer cells (excessive proliferation, impaired cell death signaling, and deregulated metabolism) [10]. In normal cells, energy transduction eventually leads to the oxidation of nutrients via oxidative phosphorylation. Glycolysis continuously generates pyruvate (Pyr), which is preferentially transported into mitochondria and further metabolized via the TCA cycle; and the ratio of NAD^+^ to NADH is balanced in favor of NAD^+^ in normal cells. While in cancer cells, a high rate of glycolysis is observed with a highly increased glucose uptake. Lactate dehydrogenase isoform A (LDHA) preferentially converts accumulating Pyr to lactate, thereby regenerating NAD^+^ from NADH to maintain glycolysis. Excess lactate is secreted and contributes to an extracellular environment that promotes tumor progression [11]. Meanwhile, the accumulation of lactate in tumors implies an increase in NADH relative to NAD^+^. For example, the intracellular NADH level in the breast cancer cell line (Hs578T) has been quantified to be approximately 1.8-fold higher than in breast normal cells (Hs578Bst) [12]. As mentioned above, The NAD^+^:NADH ratio plays an important role in regulating the intracellular redox state and several enzymes involved in the regulation of the metabolism. It has been reported that changes in NAD^+^ concentration and/or the NAD^+^:NADH ratio can induce DNA repair and increase cell defense, by regulating diverse signaling pathways and transcriptional events, and thus plays an important role in cancer progression [13,14]. For instance, NAD^+^ has been proved to regulate DNA damage repair, cell cycle progression, and epithelial-to-mesenchymal transition (EMT) via PARP-mediated ADP-ribosylation and SIRT-mediated deacetylation [15,16,17]. In addition, NAD^+^ can be metabolically converted to cADPR, a specialized signaling molecule that regulates multiple aspects of cancer biology, including cell survival, apoptosis, and inflammation [18]. Besides the NAD^+^/NADH system, another distinctive biochemical characteristic in carcinogenesis is the increased availability of the anabolic coenzyme NADPH. Cancer cells adapt their metabolism to fulfill their increased demand for energy, biosynthetic intermediates, and to counter aerobic respiration-induced oxidative stress by diverting glycolysis to pentose phosphate pathway (PPP). During this process, NADPH is produced to counteract reactive oxygen species (ROS) and to act as a cofactor for the synthesis of nucleotides, proteins, and fatty acids [19]. It has also been proved that NADK had a cancer-promoting role that converted cytosolic and mitochondrial NAD^+^ to NADP^+^, which could be further reduced to NADPH in PPP [20]. Taken together, in cancerous cells, any changes in the concentrations of these metabolites will break their synthesis and consumption homeostasis, thereby affecting the functions of their associated proteins and signaling pathways to participate in multiple processes in cancers to modulate cell metabolism, survival, progression, and invasion. To elucidate these molecular mechanisms will be of great significance for the treatment of cancer targeting NAD^+^ and its metabolites.

The mechanisms of these pyridine nucleotides to act as essential cofactors in redox reactions, or substrates in ribosyl transfer reactions, have been recently reviewed in detail [21]. Moreover, in addition to these regulatory functions, recent work has shown that NAD^+^ and its metabolites also regulate the activity of ion channels, including the Slo K^+^ channels, voltage-gated potassium channels and sodium channels, ATP-regulated K^+^ channels, and some specific types of calcium channels [8]. Considering the important role of Ca^2+^ homeostasis in malignant transformation, tumor progression, and response to treatment [22], we review in this article the evidence implicating NAD^+^ and its metabolites as regulators of calcium channels, and the function of these ion channels in cancer, aiming to shed light on the mechanisms of NAD^+^ metabolites related to calcium signaling in tumorigenesis, metastasis, and therapy. At the same time, this review is also to thank Professor Barry V. L. Potter for his great contribution in this research field over the years.

## 2. Transient Receptor Potential Melastatin 2 (TRPM2) Channel

The TRPM2 channel is a Ca^2+^-permeable cation channel [23] that functions as a polymodal channel responding to warm temperature, pH, trace metal ions, as well as ROS [24,25,26,27,28]. It is abundant in the brain, spleen, liver, lung, heart, myeloid cells, and so on. Accumulating evidence indicates that the TRPM2 channel is a complex molecular machine crosslinked with several signaling pathways, uniquely linking the adenine nucleotide metabolic network to the intracellular redox status. So it is critical to clarify the TRPM2 gating mechanisms of endogenous ligands from the NAD^+^ metabolites. Each subunit of the TRPM2 tetramer contains an intracellular N-terminal MHR domain, the typical six transmembrane domains, and a large intracellular C terminus. A series of studies has demonstrated that the NUDT9 homology (NUDT9-H) domain in the C terminus was essential for binding of NAD^+^ metabolites and thereby for activating the channel [29,30,31,32]. Recent cryo-electron microscopy (cryo-EM) studies have reported structures of the full-length human TRPM2 (*hs*TRPM2) channel as well as the non-mammalian *Nematostella vectensis* and *Danio rerio* TRPM2 channels, including the ADPR-bound state with two ADPR densities in the cleft of the MHR1/2 domain and the NUDT9-H domain, respectively [33,34,35,36,37]. Although these structural studies suggest noticeable differences or even contradictions in ADPR binding and channel gating mechanisms, activation of this channel by NAD^+^ metabolites with a pyridine nucleoside structure is well recognized.

Early studies reported that NAD^+^ itself induced a large inward current through the TRPM2 channel in endogenous-expressed immunocytes and exogenous-transfected HEK293 cells [28,38]. However, later studies showed that there was not any effect when NAD^+^ was infused into TRPM2-transfected cells [39], and argued that the stimulation of the TRPM2 channel by NAD^+^ might be attributable to contamination of a trace level of ADPR in the commercially available NAD^+^ preparations [30]. Moreover, our recent study also indicated that NAD^+^ failed to bind to the NUDT9-H domain of the *hs*TRPM2 channel by using the SPR approach [40]. Although the regulation of the TRPM2 channel by NAD^+^ remains controversial, NAD^+^ is still able to regulate the *hs*TRPM2 channel by its endogenous metabolite ADPR, since stimulation of the TRPM2 channel was likely to occur after activation of CD38 to generate ADPR from the cleavage of NAD^+^ [41].

Among these NAD^+^ metabolites, ADPR is considered to be the most potent endogenous agonist of the TRPM2 channel, with EC_50_ values of 10–90 µM [23]. Since the NUDT9-H domain in the C terminal is homologous to the NUDT9 ADP-ribose pyrophosphatase (~50% similarity), the activation of the *hs*TRPM2 channel by ADPR was originally proposed to be mediated by an enzymatic process in which ADPR bound to NUDT9-H and was converted to AMP and ribose-5′-phophate [32,42]. However, this view had been refuted by later work [43,44], with a demonstration that the *hs*TRPM2 channel does not act as a chanzyme for the lack of an ADPR-hydrolase activity. Nevertheless, the binding of ADPR to the NUDT9-H domain is believed to be essential for *hs*TRPM2 channel opening, and could be impaired upon mutations in this pocket [45,46]. Our previous study identified the key residues for ADPR binding to the NUDT9-H domain by combining homology modeling, MD simulations with functional assays [47], some of which had been verified in the cryo-EM structures of the *hs*TRPM2 channel lately [34,37].

cADPR and NAADP are two other controversial TRPM2 channel activators. cADPR was well recognized to mediate Ca^2+^ signaling pathways by binding to FKBP12.6 and modulating the function of the ryanodine receptors (RyRs), which will be discussed later in this review. However, when RyRs were specifically blocked, cADPR was still able to induce the intracellular Ca^2+^ increase in rabbit skeletal muscles, indicating RyRs-independent mechanisms that contribute to cADPR-induced Ca^2+^ responses [48]. This cADPR-induced calcium flux, in addition to RyRs, was later identified by the contribution of the TRPM2 channel [29,30,31]. These studies also confirmed the activation of the TRPM2 channel by NAADP. However, later studies had challenged those views and suggested that those earlier results might be compromised by ADPR contamination in the commercial cADPR [49] and ADPR-2′-phosphate (ADPRP, a TRPM2 agonist) contamination in the commercial NAADP [50]. Nevertheless, through a combination of surface plasmon resonance (SPR), whole-cell and single-channel patch-clamp recordings with purified cADPR, one of our recent studies had clearly demonstrated that cADPR is a bona fide activator of the TRPM2 channel [40]. To further confirm whether these NAD^+^ metabolites directly activate the TRPM2 channel, it is required to provide the evidence of the high resolution structure information in future.

As we all know, oxidative stress results from an imbalance between the amount of ROS produced and antioxidant levels. Low levels of ROS can modulate cell survival and metabolic pathways to enhance cell proliferation, while high levels of ROS damage tissues through protein oxidation, lipid peroxidation, DNA oxidation and mutagenesis that further activates cell death pathways [51]. Elevated levels of ROS have been found in the majority of cancers and promote tumorigenesis through activation of transcription factors, signaling pathways and DNA damages [52]. Under such circumstances, cancer cells show increased oxidative stress. The ADPR level and NADH/NAD^+^ ratios are also altered [53], which thus activates the TRPM2 channel. For example, in pancreatic cancer cells, SIRT6 was observed to catalyze the NAD^+^-dependent deacetylation of target histones, thereby generating 2′-*O*-acetyl-ADPR (OAADPR) that can be subsequently hydrolyzed to ADPR, which in turn activates the TRPM2 channel, triggering Ca^2+^ influx, and further to induce the expression of IL-8 and TNF, and enhance cell migration [54].

**Figure 2:**
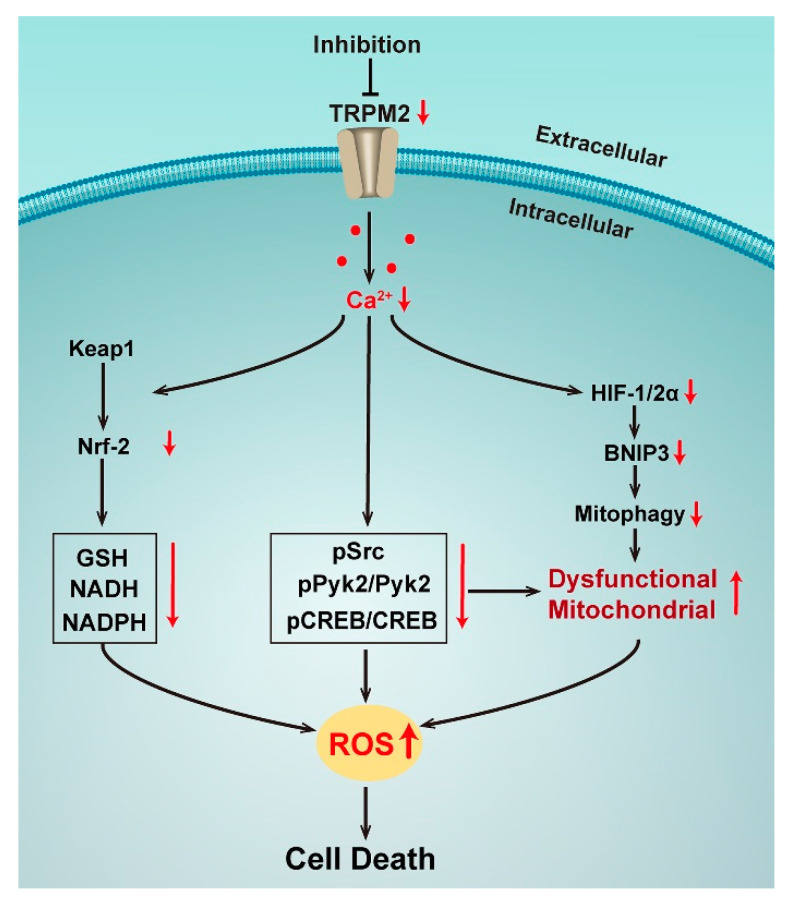
Effects of TRPM2 inhibition in neuronblastoma.

In most of the nonmalignant cells, it is supported that a sustained increase in intracellular Ca^2+^ or Zn^2+^ may occur leading to cell death simultaneously with the TRPM2 activation by oxidative stress [55,56,57]. However, the data in cancer models mostly supports the conclusion that TRPM2 expression and function have an important role in preserving cancer cell viability and survival. Consistent with this view, the TRPM2 channel has been found to be highly expressed in numerous cancers including bladder, breast, head and neck, lung, pancreatic, prostate, melanoma, and neuroblastoma [51], among which most studies were focused on the neuroblastoma [58,59,60,61,62,63,64] (Figure 2). The higher levels of ROS in cancer cells impel the enhancement of their anti-oxidant capacity to detoxify ROS and preserve cells viability. The transcription factor nuclear factor (erythroid-derived 2)-related factor-2 (Nrf2) takes responsibility for expression of a series of genes to regulate enzymes or cofactors involved in the anti-oxidant response [65]. Nrf2 has been observed to be highly expressed in many malignant cells, and regulated by Ketch-like ECH-associated protein 1 (Keap1) via a Ca^2+^-dependent process [66,67]. Meanwhile, it has been found that with the inhibition of the TRPM2 channel, Ca^2+^ influx was reduced, which caused the reduction of Nrf2. Its downstream enzymes involved in GSH, NADPH, and NADH production were significantly decreased, which led to weakened antioxidant responses, increasing the susceptibility to chemotherapeutic agents and decreasing cell survival and tumor growth [68].

In addition to Nrf2, the transcription factors HIF-1/2α and cyclic AMP-responsive element binding protein (CREB) are also regulated by TRPM2. HIF-1/2α was significantly reduced with the TRPM2 inhibition by expression of the negative short splice variant TRPM2-S in neuroblastoma cell lines. ROS thus increased as well as the accumulation of dysfunctional mitochondria with a reduced bioenergetic capacity by the down-regulation of autophagy/mitophagy via a decreasing mitochondrial membrane potential, and impairing Ca^2+^ uptake [59]. A study has further confirmed that re-expression of wild type TRPM2 in such a condition could rescue cell viability, mitochondrial function, and reduce ROS, demonstrating the critical role of TRPM2-mediated Ca^2+^ entry in the modulation of tumor growth, mitochondrial function, and cellular bioenergetics in neuroblastoma [60]. Interestingly, this mechanism was also found to be related to TRPM2-mediated CREB expression. CREB is a key transcription factor that regulates the genes involved in oncogenesis and cell survival. Ca^2+^ influx via TRPM2 results in the activation of phosphorylation of Pyk2, which regulates the cell survival and tumor growth of various cancers through the CREB pathway, leading to increased expression of phosphorylated and total CREB. When TRPM2 was inhibited, pPyk2, Pyk2, pCREB, and CREB were reduced and mitochondrial function as well as mitochondrial Ca^2+^ uptake were impaired, together with more mitochondrial and cellular ROS, reducing cells survival and tumor growth [62]. Taken together, all studies in neuroblastoma show the critical role of TRPM2 that modulates both ROS production and the antioxidant response through the Ca^2+^ entry via the channel activation. When the TRPM2 channel is inhibited, ROS are significantly increased by both mitochondrial dysfunction and reduced antioxidants, then reaching to a cytotoxic threshold of cell death.

In other types of cancers, a high expression of TRPM2 was also observed to increase the cancer cells survival and proliferation. The mechanisms might include minimizing DNA damage in breast adenocarcinoma cells [69,70]; increasing the migration/invasion of pancreatic ductal adenocarcinoma cells [71], gastric cancer cells [72], and tongue carcinoma SCC cells [73]; or inhibiting nuclear ADP-ribosylation in prostate cancer cells [74]. It has also been reported that the inhibition of TRPM2 could accelerate the cancer cells death by increasing the intracellular ROS in non-small cell lung (NSCLC) cells [75]; the impairment of autophagy through the JNK-signaling in gastric cancer cells [76]; or reducing the G2/M ratio in the proliferation cycle of leukemia cells [77] and NSCLC cells [75]. In addition, we also noticed that recent studies have reported a novel long non-coding RNA TRPM2-AS with a high expression correlated with a larger tumor size, advanced TNM stage, and poor patient outcomes in a variety of cancers [78,79,80,81,82,83,84,85,86,87]. Since its mechanisms in tumors are complicated and less related to the regulation of the signaling pathway by the ion channel, we did not review it here. Further work will be necessary to understand its impact on TRPM2 expression and function, as well as its role in tumorigenesis.

However, a few studies had found that TRPM2 high expression in certain types of cancer cells correlated with improved patient outcomes. For example, TRPM2 overexpression promoted apoptosis of T24 bladder cancer cells [88]; survival time was significantly longer in patients with higher TRPM2 levels than in those with lower TRPM2 levels [89]. These data suggest that the differential effects of the TRPM2 channel in cancers depend on the types of carcinomas. We believe that the oxidative stress balance regulated by the TRPM2 channel via NAD^+^ metabolites is the decisive factor of this channel to be a friend/foe. In any case, the NAD^+^ and its metabolite-activated TRPM2 channel is an exciting potential therapeutic target for a variety of cancers where the mechanisms in tumorigenesis, metastasis, and therapy need to be further uncovered.

## 3. Two-Pore Channels (TPCs)

Two-Pore Channels (TPCs, *TPCN* as the gene is named) are cation permeable channels located on endolysosomal membranes and act as important mediators of intracellular Ca^2+^ signaling. They are subdivided based on their structural similarity into three groups: TPC1, TPC2, and TPC3. These channels contain two putative pore-forming repeats, and each of these repeats contains six transmembrane segments and an intervening pore-loop, an architecture common to numerous voltage-gated ion channels. The transmembrane regions of TPCs are homologous to that of Na_v_ or TRP channels [90]. However, unlike these related channels, TPCs are not expressed on plasma membranes. Only TPC1 and TPC2 are found in human and mouse cells. TPC1 is found in a range of endolysosomal organelles, and TPC2 is the predominant form expressed in late endosomes and lysosomes [91]. It has been observed that cells expressing the TPC2 channel showed a marked calcium release on intracellular application with NAADP, while genetic knockdown of this channel abolished NAADP-induced calcium release, indicating that TPC2 is an endogenous target of NAADP [92]. This study also proved that membranes enriched with TPC2 exhibited a high affinity for NAADP binding. Besides, it had proposed that NAADP was the most potent calcium regulator of NAD^+^ metabolites, since it stimulates calcium release at concentrations as low as 5 to 10 nM. However, intracellular dialysis of 1 mM NAADP failed to elicit a Ca^2+^ release, indicating homologous self-inactivation of the Ca^2+^ release process by NAADP, which prompted that a high-affinity binding site on the TPC2 may confer a channel opening, while a low-affinity site may confer inactivation/desensitization [93]. In addition to TPC2, NAADP also evokes endolysosomal cation release via TPC1 [94], and it has shown that arginine residues in the first S4–S5 linker were required to trigger Ca^2+^ signaling upon NAADP binding to TPC1 [95]. However, a recent study provided information that human TPC is, in fact, not directly activated by NAADP [96]. Although the 3D structures of mouse TPC1 and human TPC2 were recently determined by cryo-EM [97,98], the bound state of NAADP on these channels remains unknown, and further investigation is still required. There are also a series of studies developing a photoaffinity probe for the NAADP receptor, 5-N_3_-NAADP, which showed that NAADP did not bind to TPCs directly, but through NAADP-binding proteins [99]. It is worth mentioning that in addition to TPCs, NAADP also regulates RyRs and the TRP subtype mucolipin 1 (TRPML1) (this will be discussed later in this review) and the TRPM2 channel (see above). Therefore, it has been suggested that the responses of multiple NAADP targets are integrated such that the release of calcium by NAADP via TPCs is amplified by those neighboring receptors to generate well-orchestrated calcium oscillations [99,100].

**Figure 3:**
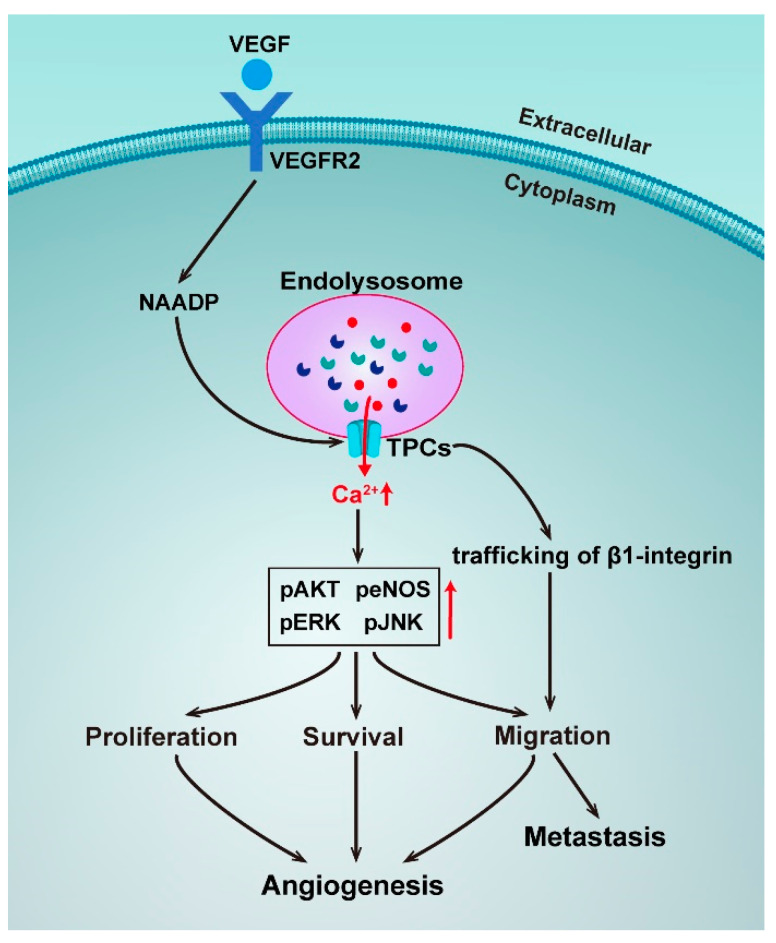
Schematic representation of the roles of TPCs in angiogenesis and metastasis.

The TPCs have been reported to be involved in various pathophysiological processes, including cell growth and development, metabolism, and cancer progression [101]. For instance, TPC2 was observed to be overexpressed in oral squamous cell carcinoma cell lines, raising intriguing questions regarding the role of TPC2 as a driver of oncogenesis [102]. In addition, *TPCN2* was found to be significantly associated with survival in bladder cancer [103], and has also been reported to be one of the six gene signatures correlated with prostate cancer to predict postoperative biochemical recurrence [104]. Many studies on the association between TPCs and cancers have revealed the role of NAADP/TPC/Ca^2+^ signaling (Figure 3).

Angiogenesis is a process that is crucial for cancer progression and a key step in the transition of a tumor’s state from benign to malignant. Vascular endothelial growth factors (VEGF) are the major regulators of angiogenesis and regulate endothelial cells with cell proliferation, migration, and sprouting in the early stages of angiogenesis [105]. A novel pathway for VEGF signaling transduction had been reported, such that VEGF receptor activation led to NAADP and TPC2-dependent Ca^2+^ release from acidic Ca^2+^ stores, which in turn controlled angiogenic response. Pharmacologically using the NAADP antagonist Ned-19 or genetically using *TPCN2*^−/−^ mice, it was found to dramatically reduce angiogenic responses to VEGF in vitro and in vivo [106]. The same mechanism was later confirmed by a study using the natural product Naringenin to inhibit the VEGF-induced angiogenesis [107]. An implication of these studies is the possibility to target TPC2 to develop anti-angiogenics as a strategy for cancer treatment.

Uncontrolled cell proliferation is another basic feature of cancers. A recent study has discovered that NAADP-induced Ca^2+^ release was blocked by genetic silencing of TPC1, and a pharmacological and genetic blockade of TPC1 dramatically reduced fetal bovine serum (FBS) and induced Ca^2+^ release and proliferation of metastatic colorectal cancer (mCRC) cells established from liver metastasis of human patients, thereby hinting at TPC1 being a novel therapeutic target in mCRC patients [108]. Metastatic invasion is the major cause of cancer-related deaths. A study has unveiled that TPCs played a crucial role in the formation of metastasis, as silencing TPC1 and TPC2 reduced the adhesion and migration of invasive tumor cells in vitro, and pharmacological TPC inhibition and siRNA silencing of TPC2 reduced the formation of lung metastasis in vivo [109]. However, in this study, the activation of TPCs by NAADP was not directly proved, suggesting only an involvement of trafficking of β1-integrin, a protein that is prominently involved in tumor migration. Take into account the important role of Ca^2+^ homeostasis in β1-integrin trafficking and the molecular mechanisms of NAADP-induced TPC activation, the TPC-mediated Ca^2+^ signaling in the metastasis process should be independently resolved.

Besides, there are some studies on the functions of TPCs in other physiological processes related to tumorigenesis. For example, NAADP-evoked Ca^2+^ signals through TPC1 and TPC2 sustained glutamate-induced autophagy in SHSY5Y neuroblastoma cells [110]. Silencing of TPC2 attenuated epidermal growth factor-induced vimentin expression in MDA-MB-468 breast cancer cells [111]. TPC2 overexpression in 4T1 mouse breast cancer cell lines and human HeLa cervical cancer cell lines inhibited the fusion of autophagosomes and lysosomes, causing the accumulation of autophagosomes [112]. Besides, TPC2 overexpression led to the evocation of the defects of pigmentation that is closely related to the development of melanoma, and its interactivity with Rab GTPases underpinned NAADP-evoked Ca^2+^ signals [113]. Taking all of the abovementioned together, TPCs are druggable targets that can interfere with tumorigenesis, angiogenesis, and metastasis.

## 4. Ryanodine Receptors (RyRs)

The RyRs represent another class of calcium channel with the regulation by NAD^+^ and its metabolites. RyRs are located on the sarcoplasmic and endoplasmic reticulum (SR/ER) forming a series of intracellular Ca^2+^ channels. There are three major structurally similar RyRs mammalian isoforms: RyR1, RyR2, and RyR3. RyR1 and RyR2 are the major RyR isoforms in skeletal and cardiac muscle, respectively, and RyR3 is expressed in various tissues along with the other two isoforms [114]. Dating back to 1994 and 1995, studies have found that NAD^+^ could increase the open probability of RyR1 and RyR2 [115,116]. It was then reported that RyR1 contained several dehydrogenase and NAD^+^/NADH oxidoreductase domains, and some residues that participate in NADP^+^ binding in isocitrate dehydrogenase were conserved in RyR1, suggesting that the channel may be capable of binding to NAD^+^ metabolites [8,117]. Later, single channel recordings from RyRs incorporated into lipid bilayers revealed that NADH (2 mM) inhibited the activity of RyR channels by 84% in permeabilized rat ventricular myocytes [118]. These results all suggest that NAD^+^/NADH is the direct modulator of RyRs.

Later on, more studies have focused on the activation of RyRs by cADPR. It was first proposed that cADPR could activate RyRs in 1991 [3]. The abovementioned studies in 1994 and 1995 also suggested similar conclusions [115,116]. All three RyR isoforms have been shown to mediate cADPR-induced Ca^2+^ release [119,120]. One of our own pieces of research, using a caged cADPR analogue, also confirmed its activation on RyR2 and RyR3 in Jurkat T cells [121]. However, evidence regarding whether cADPR acts directly on the receptors is lacking. RyR was isolated from the cellular environment and incorporated into artificial membranes under voltage-clamp conditions, which could avoid confounding cellular factors and decide the direct interaction of cADPR on the RyR channel. Numerous studies have found that cADPR had no effect on the gating of all the three types of RyRs [122]. These results suggest that cADPR may not act directly on RyRs, but via some accessory proteins to activate RyRs. Two cADPR-binding proteins—140- and 100-kDa proteins—have been identified in sea urchin egg homogenates by 8-N_3_-cADPR, an analog of cADPR, as a photoaffinity probe [123]. Moreover, calmodulin-dependent protein kinase II (CaMKII), calmodulin, and FK506-binding protein, FKBP12.6 have been shown to be required for cADPR action [124,125,126]. A recent study by synthesizing a novel photoaffinity labeling cADPR agonist identified glyceraldehyde 3-phosphate dehydrogenase (GAPDH) as one of the bridging proteins between cADPR and RyRs [127].

Besides cADPR, involvement of RyRs for NAADP-activated Ca^2+^ mobilization has also been evidenced. For instance, NAADP induced Ca^2+^ release from rat heart microsomes, and RyR2 activated by NAADP from dog heart incorporated into bilayer lipid membranes were observed [128]. In addition, nanomolar concentrations of NAADP triggered Ca^2+^ release from skeletal muscle SR, which was due to a direct action on RyR1, since NAADP increased the open probability of the purified RyR1 channel by using a single-channel recording [129]. Another series of studies showed that co-injection of the RyRs antagonists ruthenium red with NAADP abolished the Ca^2+^ signal from NAADP, and Jurkat T cells with largely reduced expression of RyRs did not respond to microinjections of NAADP, suggesting that the Ca^2+^ release channel sensitive to NAADP in T-lymphocytes is the RyRs [130,131]. However, none of these results had demonstrated the direct activation of RyRs by NAADP. A more recent study using combinatorial knockouts and antibodies against RyRs and TPCs compared their relative contribution to NAADP-induced Ca^2+^ release from permeabilized pancreatic acinar cells. It was observed that, with a sequence of RyR1 > TPC2 > RyR3 > TPC1 >> RyR2, and the primary, but very small, NAADP-elicited Ca^2+^, release via TPCs triggered the detectable Ca^2+^-induced Ca^2+^ release (CICR) via RyRs occurring from the granules and the ER [132].

Although further investigations are required to prove the molecular mechanisms of direct/indirect activation of RyRs by NAD^+^ and its metabolites mentioned above, their regulations on RyRs that involved in the physiological and pathological processes such as cancer development are well established. For example, in Namalwa B lymphoma cells, ryanodine stimulation of Ca^2+^ release decreased both CD38 protein abundance and cyclase activity, indicating a negative feedback mechanism between the RyRs channel and CD38, which could directly affect the signaling pathways of NAD^+^ metabolites catalyzed by CD38 [133]. Oxygen and glucose deprivation (OGD) due to insufficient blood circulation can decrease cancer cell survival and proliferation of solid tumors with the activation of adenosine 5′-monophosphate-activated protein kinase (AMPK). One study found that NAD(P)H: quinone oxidoreductase 1 (NQOD1) played a key role in the AMPK-induced cancer cell death in OGD through the CD38/cADPR/RyR/Ca^2+^/CaMKII signaling pathway [134]. Besides, there are also some reports that showed a high diversity of the RyRs expression in tumors. For example, in a research including 57 ductal, human breast cancer specimens, moderate-to-high expression of RyRs by immunostaining was found in 82% of the specimens, and there was a direct correlation between RyRs levels and tumor grades [135]. RyR2 was over-expressed in melanoma tissues [136], and RyR3 over-expression was detected in breast cancer [137]. However, in comparison with normal thyroid tissues, tissues derived from thyroid carcinoma exhibited decreased expression of RyR2, which was tightly associated with lymphatic metastasis, extracapsular extension, and the TNM stage [138]. Many studies have also reported the differential regulations of RyRs in the proliferation or migration of different types of cancer cells [139,140,141,142], but various functions of RyRs in respectively characterized malignant diseases are still needed to be clarified, especially the roles of NAD^+^ and its metabolites in the Ca^2+^ signaling pathway regulated by RyRs.

## 5. Transient Receptor Potential Channel Subtype Mucolipin 1 (TRPML1)

The transient receptor potential channel subtype mucolipin 1 (TRPML1) is an integral part of the acidic vesicles in the endolysosomal system. Similar to all TRP channels, each TRPML1 is composed of 4 subunits which possess 6 transmembrane spanning domains with cytosolic N and C terminals. It is widely distributed within the later vesicles of the endocytic pathway [143,144]. Research that fused the TRPML1 into lipid planar bilayers observed that NAADP activated Ca^2+^ release at concentrations of 1–1000 nM, and TRPML1 gene silencing reduced the extent of this NAADP-sensitive Ca^2+^ release. In addition, the blockade of TRPML1 by anti-TRPML1 antibodies almost abolished NAADP-induced activation of lysosomal Ca^2+^ channels, which provided the direct evidence to show that a NAADP-sensitive Ca^2+^ release is characteristic of the TRPML1 channel [145,146]. Research from the same group further claimed that NAADP-induced activation of the TRPML1 channel could not be observed in lysosomes from TRPML1^−/−^ cells, but was restored by re-expressed TRPML1 into these cells. This work has also proved that NAADP regulated TRPML1 activation via promoting the interaction of endosomes and lysosomes, and thereby regulated lipid transport to lysosomes [147]. However, some studies put forward a contrary view that TRPML1 was not the target for NAADP, because neither overexpression of TRPML1 nor the dominant negative TRPML1 mutant D471K affected the NAADP-mediated Ca^2+^ signals [148]. However, there was a comment on the above research that proposed that a direct recording of lysosomal TRPML1 currents or measurements of Ca^2+^ release from lysosomes are needed for solid evidence to specify the TRPML1 channel as a NAADP-sensitive lysosome sensor [149].

Up to now, no evidence has been presented on the involvement of TRPML1 activated by NAADP in malignant transformation. There are only limited studies on the function of TRPML1 in cancer cell proliferation. For example, the increase of TRPML1 expression attenuated MAPK and mTORC1 signaling to sustain macropinocytosis, and avoid proteotoxic stress among melanoma cells [150]. TRPML1 maintains oncogenic mutations in the RAS family by mediating cholesterol de-esterification and transport, and reducing the proliferation of cancer cells that express oncogenic mutations by TRPML1 inhibition [151,152]. TRPML1 was specifically upregulated in triple-negative breast cancer (TNCB), which regulates TNBC development through controlling mTORC1 activity and the release of lysosomal ATP, while genetic downregulation or pharmacological inhibition of TRPML1 suppressed the growth of TNBC [153]. The functions of TRPML1 channels in tumorigenesis, especially the roles of NAD^+^ metabolites, still require further elucidation.

## 6. Conclusions and Expectation

**Figure 4:**
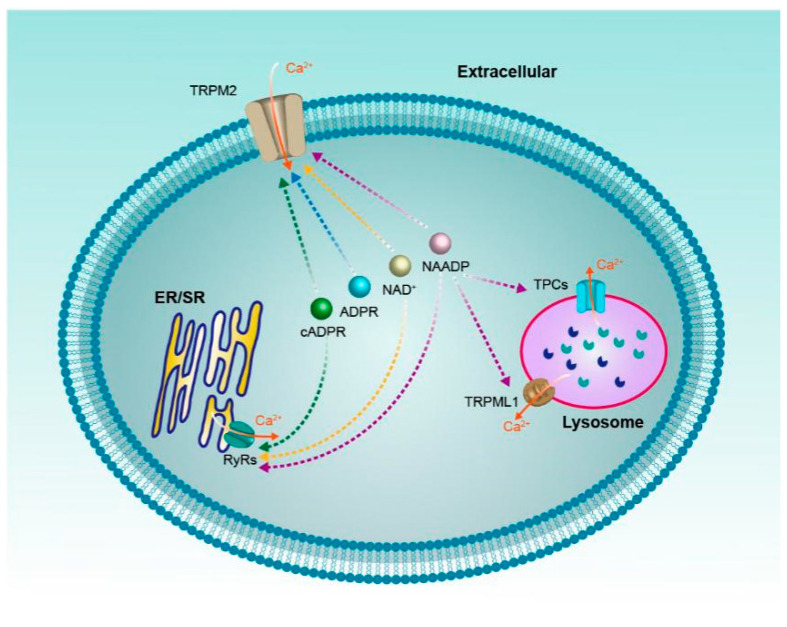
NAD^+^ and its metabolites (ADPR, cADPR, NAADP) regulate calcium channels.

In classical biochemistry, NAD^+^ and its metabolites are most frequently viewed as soluble electron carriers. Recent research suggests that these metabolites can also regulate cell signaling by acting as the modulator of ion channels, just like the calcium channels reviewed above (Figure 4). It should be noted that these ion channels often have multiple activators/regulators, for instance, RyRs can be activated by a variety of NAD^+^ metabolites, even the non-NAD^+^ metabolites ryanodine and caffeine. On the other hand, one NAD^+^ metabolite can also regulate various calcium channels, the NAADP for example, which may regulate all the four ion channels mentioned above. Moreover, since NAD^+^ metabolites are a mutual conversion and the concentrations of NAD^+^ metabolites change dynamically in vivo, it is important for us to comprehensively investigate how the contributions from different activators/regulators of these ion channels mediate intracellular Ca^2+^ signaling in the complicated tumor pathogenesis in future.

Professor Potter B.V.L. has made brilliant contributions to the identification of these NAD^+^ metabolites as these ion channels activators/regulators, which have been cited above. More importantly, based on the fact that these ion channels have been regarded as new cancer therapeutic targets, Professor Potter has designed and synthesized a series of ligands/inhibitors of these calcium channels [72,154,155,156,157,158,159,160,161,162,163,164,165,166,167,168], which certainly play critical roles in the development of anti-cancer drugs targeting these ion channels. In addition, some potassium and sodium channels can also be activated by NAD^+^ and its metabolites, and there are also some calcium channels whose activity can be indirectly affected by NAD^+^ and its metabolites. For example, the P2 × 7 channel is activated by ADP-ribosyltransferase 2.2-dependent ADP ribosylation in the presence of extracellular NAD^+^ [169]. Moreover, the CD38-cADPR-RyRs signaling pathway modulates store-operated calcium entry through transient receptor potential ion channels (TRPCs) [170], which was also evidenced by co-immunoprecipitation of RyRs and TRPC3 [171], and gating of the TRPC under activation of RyRs [172]. All of these NAD^+^ metabolite-regulated ion channel-mediated signaling pathways have been increasingly demonstrated to play important roles in tumorigenesis, metastasis, and therapy. This field is still maturing, and is surely going to open doors to more exciting studies in the future.
